# Acknowledgement to Reviewers of *Marine Drugs* in 2016

**DOI:** 10.3390/md15010015

**Published:** 2017-01-11

**Authors:** Marine Drugs Editorial Office

**Affiliations:** MDPI AG, St. Alban-Anlage 66, 4052 Basel, Switzerland; marinedrugs@mdpi.com

The editors of *Marine Drugs* would like to express their sincere gratitude to the following reviewers for assessing manuscripts in 2016.

We greatly appreciate the contribution of expert reviewers, which is crucial to the journal’s editorial process. We aim to recognize reviewer contributions through several mechanisms, of which the annual publication of reviewer names is one. Reviewers receive a voucher entitling them to a discount on their next MDPI publication and can download a certificate of recognition directly from our submission system. Additionally, reviewers can sign up to the service Publons (https://publons.com) to receive recognition. Of course, in these initiatives we are careful not to compromise reviewer confidentiality. Many reviewers see their work as a voluntary and often unseen part of their role as researchers. We are grateful to the time reviewers donate to our journals and the contribution they make.

If you are interested in becoming a reviewer for *Marine Drugs*, see the link at the bottom of the webpage http://www.mdpi.com/reviewers.

The following reviewed for *Marine Drugs* in 2016:
Aarstad, Olav AndreasLaBarbera, Daniel V.Abe, HidekiLabes, AntjeAfiyatullov, Shamil Sh.Lalk, MichaelAfonso, Clélia N.Lang, SiegmundAgyei, DominicLaparra, MoisésAiba, TetsuyaLau-Cam, Cesar A.Aki, TsunehiroLauder, BobAlber, Birgit E.Laurienzo, PaolaAl-Horani, RamiLauritano, ChiaraAllen, EricLe Pogam, PierreAllsopp, PhilipLeão, Pedro N.Alo, RaffaellaLee, Bong HoÁlvarez, MercedesLee, Che-HsinAlves, CelsoLee, Chia-hungAmsler, CharlesLee, Ji-youngAnadon, ArturoLee, Jun SikAnas, Andrea RoxanneLee, KyubockAndreadou, IoannaLee, Tsung-HanAndrianasolo, EricLee, VictorAnesi, AndreaLee, Yeon-JuAnraku, MakotoLeone, AntonellaArai, RyoichiLi, NingAranaz, InmaculadaLi, YanAranda-Rodriguez, RocioLiakos, Ioannis L.Arduini, AlessandroLiang, Yu-chihArnason, JohnLiaw, Chih-ChuangArnold, EvaLicea Navaro, Alexei FedorovishAvila, ConxitaLin, Hai-shuAviles, Francesc X.Lin, Hsi-hsienAzuma, KazuoLin, ZhenjianAzzimonti, BarbaraLiou, Chian-JiunBae, Hae-RahnLittle, R. DanBaker, BillLiu, JunyingBanack, SandraLiu, PinghuaBanerjee, MonimoyLo Giudice, Angelina LoBanerjee, PratikLopanik, Nicole B.Banskota, Arjun H.Louzao, M. CarmenBaraka, KhaledLu, JunBarbosa, MarianaLu, Mei-ChinBarnathan, GillesLu, SizhaoBarreira, LuísaLüdeke, SteffenBasini, GiuseppinaLuesch, HendrikBasti, LeilaLynch, JenniferBates, Roderick W.M, Valan ArasuBeach, DanielM. Greene, CatherineBeaulieu, LucieMacMillan, JohnBelas, RobertMaderna, AndreasBelovich, Joanne M.Madhukar, BurraBenkendorff, KirstenMaeda, HayatoBerecki, GezaMajumder, KaustavBerrue, FabriceMancini, InesBerry, JohnManganiello, GelsominaBianco, Armando DorianoMangoni, AlfonsoBianco, I.D.Marino, AngelaBjørndal, BodilMartin, JessicaBlanco, JuanMartin, Luc J.Blandino, GiovanniMartínez-Alvarez, OscarBlunt, JohnMarx, FlorentineBoateng, Joshua S.Mass, TaliBoddy, Christopher N.Matsunaga, ShigekiBøgwald, JarlMatsuura, HiroshiBöhm, VolkerMaupin, C. MarkBondon, ArnaudMazur-Marzec, HannaBoquet, DidierMcCall, Jennifer R.Bose, UtpalMcCormick, RachelBoussiba, SammyMcMullin, David R.Boustie, JoelMeadus, William JonBovee, Toine F.H.Mebs, DietrichBrecker, LotharMedina, IsabelBreuss, JohannesMenna, MarialuisaBrinkman, DianeMetcalf, JamesBrown, LindsayMEUNIER, BernardBrunel, JeanMeyer, Anne SBugni, TimMiert, Sabine VanBumgardner, Joel DMilthorpe, BruceBurkhart, David J.Mishra, NigamCahoon, LawrenceMiyaoka, HiroakiCalado, RicardoMiyashita, KazuoCaldwell, GaryMiyazaki, YasunoriCallery, Patrick S.Mizuno, MasashiCampos, AlexandreMoffit, Michelle C.Cañavate, JoséMohammad, AltafCapon, RobertMölleken, HelgaCARAMELLA, CARLAMonti, Maria ChiaraCarbone, AnnaMorabito, MarinaCarmeli, ShmuelMorales, DianaCarrano, LuciaMoran, YehuCasapullo, AgostinoMoreno, Antonio D.Caulier, GuillaumeMorfini, GerardoCerón-García, María Del CarmenMori, NaokiCesaro, AttilioMorimura, ShigeruChang, Hsueh-WeiMossialos, DimitrisChang, Yi-tangMotti, CherieChao, Louis KuopingMüller, WernerChauvierre, CédricMüller, Werner E.G.Chen, GuoxunMundt, SabineChen, HaixiaMuramoto, KojiChen, Jiann-ChuMurch, SusanChen, Jih-JungMurphree, S. ShaunChen, LihuaMurphy, Patrick J.Chen, Shiu-NanMuzzarelli, Riccardo A. A.Cheng, Yuan-BinNaderer, ThomasCherian, PhilipNánási, PéterCheung, Randy Chi FaiNasopoulou, ConstantinaChiang, Meng-tsanNasri, RimChiesa, GiuliaNavarro, Juan CarlosChi-Feng, HungNazar, HamdeChinchilla, RafaelNelson, DavidChiou, Ya-LingNewman, DavidChiu, Tsai-HsinNicoletti, RosarioCho, HyunahNishimura, ShinichiChoi, HyukjaeNisticò, RobertoChoi, Jae SueNoh, Jeong SookChoi, Jong-ilNorton, RaymondChong, Parkson Lee-gauNovakovic, KatarinaChou, JoshuaNunes, CláudiaChristensen, LarsO'Doherty, GeorgeChruszcz, MaksymilianOh, Dong-ChanChun, Byung-sooOh, Jung HwanChynoweth, David P.Okunade, Adewole L.Ciavatta, M. LetiziaOrtiz, AbelCleary, Margot P.Osaki, TomohiroColliec-Jouault, SylviaOsborn, HelenColombo, DiegoOzeki, YasuhiroConstantino, ValeriaPae, Ae NimCosta, MonyaPan, Cheol-hoCosta, Pedro ReisPattenden, GeraldCostantini, MariaPaulsen, Berit SmestadCostantini, SusanPautz, AndreaCoustau, ChristinePavon-Djavid, GracielaCouzinet-mossion, AureliePeigneur, SteveCrespo, Mariano SánchezPereira, José AugustoCruz-Rivera, EdwinPereira, LeonelCustódio, LuísaPerez, Maria JoseD’Orazio, NicolantonioPerez-Jimenez, JaraDai, MingjiPerillo, EmilianaDaly, NorellePescitelli, GennaroDavid, Betancur-anconaPetit, KarinaDe Agostini, ArianePetkuviene, JolitaDe Rosa, Maria CristinaPicot, L.Defoirdt, TomPiganelli, JonDenny, BillPiggott, AndrewDharmaraj, SelvakumarPillay, VinessDi, LiPinheiro, Maria Lúcia BelémDijkstra, Bauke W.Pinto, Diana C. G. A.Dioum, Elhadji M.Plaza, AlbertoDucancel, FrédéricPleissner, DanielDufossé, LaurentPouchus, Yves-FrancoisDuh, Chang-YihProksch, PeterDuverger, EricPucciarelli, SandraDyshlovoy, SergeyQaralleh, Haitham N.Ebrahim, HassanQiu, HuanEbrahim, WeaamQuesada, AntonioEdirisinghe, MohanQuinn, Mark T.Edrada-Ebel, RuangelieQuinn, RonEgan, SuhelenQvit, NirEkuni, DaisukeRaabe, GerhardElmore, Donald E.Raatz, Susan K.Esteves, Ana CristinaRadovic, Jagos R.Evans, FranklinRafael, DianaEvin, GenevièveRahman, AzizurFam, BarbaraRaina, Jean-BaptisteFassouane, AzizRämä, TeppoFatouros, DimitriosRamadan, UsamaFaulkner, SimonRamos, Adrián M.Feng, YuRao, ChristopherFernández, José J.Raposo, Maria Filomena JesusFernandez, StefanRasenick, MarkFernando, Rodríguez-SerranoRebollo, AngelitaFerreira, IsabelRebuffat, SylvieFerrieres, VincentRein, KathleenFestuccia, ClaudioReitzel, AdamFigueroa, Félix LópezReynisson, JóhannesFink-Gremmels, JohannaRho, Jung-RaeFitton, HelenRinaudo, MargueriteFleury, YannickRinge, DagmarFoley, Sarah A.RIzzo, Angela MariaFoubelo, FranciscoRobertson, AndrewFranco, ChrisRoh, ChanghyunFrankenberg Dinkel, NicoleRoh, Seong-SooFreitas, AnaRokstad, Anne MariFreitas, Ana C.Romano, GiovannaFrolova, LiliyaRosen, JohanFujii, IkuoRoullier, CatherineFujii, MikioRoussis, VasileiosFujii, RitsukoRudd, DavidFujita, MasakiRuiz-Rodríguez, MagdalenaFujita, MorifumiRussell, Fraser D.Funane, KazumiRusso, Gian LuigiFuwa, HaruhikoRustad, TuridGammone, MARyu, BomiGarces, EstherSabatier, Jean-MarcGarcia Camacho, F.Sadamoto, HisayoGarcía-Mauriño, SofíaSakai, RyuichiGavagnin, MargheritaSalanoubat, MarcelGavenonis, JasonSantos Costa De Morais, Rui ManuelGenovese, GiuseppaSantos, AbelGhoneum, MSantos, SusanaGiannaccini, MartinaSarojini, VijayalekshmiGillis, Eric P.Sass, HenrikGirgih, Abraham T.Sasso, SeverinGiunchedi, PaoloSato, KenjiGlaser, KeithSatomi, YoshikoGlukhov, AlexeySchenk, Peer M.Gnavi, GiorgioSchmalz, Hans-GuentherGnuzzo, GenoveffaSchroeckh, VolkerGobe, Glenda CSchroeder, Christina I.Gomes, Nelson G.M.Sebestik, JaroslavGozes, IllanaSenatore, AdrianoGozzo, FrancoSeo, Jung-KilGray, Christopher A.Shaw, LindseyGraziano, GuellaShenawy, Nahla S. ElGreen, Brad R.Shepherd, Suzanne M.Green, David WSheu, Jyh-HorngGribble, GordonShin, HeeGribble, Gordon W.Shin, JongheonGross, StevenShindo, KazutoshiGrothaus, Paul G.Sidorenko, AlexanderGsell, AlenaSieiro, CarmenGuarnieri, Michael T.Sigurjonsson, Olafur E.Gueven, NuriSilva, ArturGuillou, HervéSim, Sang JunGushina, IrinaSimpson, TomGutiérrez, MarcelinoSmith, AlanGverzdys, TomasSöderhäll, IreneHäder, Donat P.Solaiman, Daniel K.Y.Hadwiger, LeeSosa, SilvioHaertle, ThomasSoueidan, AssemHaglund, PeterSpahn, ViolaHamann, MarkSreedasyam, AvinashHampel, DanielaStoskopf, MichaelHansen, EspenSugahara, TakuyaHara, HiroshiSuleria, Hafiz Ansar RasulHarder, TilmannSumi, ShoichiroHardy, JoanSung, H.H.Hasegawa, YasushiSung, Ping-JyunHatae, NoriyukiSutherland, John B.Haug, TorSutherland, MarkHenriques, SoniaSuzuki, IwaneHernández-Ledesma, BlancaTadahiro, SuzukiHernandez-Prieto, Miguel A.Tahul, Maria B BadiaHerraiz, T.Takahashi, DaisukeHewage, Himaya Siddhihalu WickramaTakahashi, KoretaroHidari, KazuyaTakaichi, ShinichiHildebrand, MarkTanaka, TsuyoshiHill, Robert A.Tarman, KustiariyahHoffman, Angela M.Tartaglione, LucianaHonecker, FriedemannTepe, Jetze J.Hopkinson, BrianTeta, RobertaHosokawa, MasashiThomas, FrançoisHosokawa, SeijiroThomas, JimHu, HuijuanThomas, Olivier P.Huang, Chun-YungThoo-Lin, Paul KongHuang, Tse-HungThurber, Greg M.Hung, AndrewTokuda, MasaharuHwang, Pai-AnTommonaro, GiuseppinaHyun, Jin WonTomoda, HiroshiIanora, AdriannaTopakas, EvangelosIbeanu, GordonTrincone, AntonioIbrahim, El-SherbinyTsodikov, OlegIbrahim, Hisham R.Tsuda, MasashiIbrahim, SalamTurk, TomIndiveri, CesareTutino, Maria LuisaIoannou, EfstathiaUbhayasekera, Sarojini J. K. A.Ishigami, KenUlanova, DanaIshmael, Jane E.Umeyama, AkemiIsnansetyo, AlimUstyuzhanina, Nadezhda EItabashi, YutakaVale, PauloIto, TakuyaValentao, PatríciaIzzotti, AlbertoValério, ElisabeteJ Martín-Rodríguez, AlbertoVan Berkel, WillemJain, Mahendra KumarVan Lanen, StevenJansen, RolfVapaatalo, HeikkiJayakumar, RangasamyVarela-Álvarez, ElenaJeon, You JinVarese, Giovanna CristinaJimbo, MitsuruVázquez, J. A.Jin, Dong-HoonVeiga, María-DoloresJohn, Ho Wing ShingVieira, HelenaJohnson, John AVílchez, CarlosJoo, Hong-GuVogt, Miriam AnnikaJun, Joon-YoungVoigt, OliverJung, Jee HyungVon Schacky, ClemensKaas, QuentinVoorspoels, StefanKalinin, Vladimir IWang, ChangyunKaludercic, NinaWang, CuihuaKanakkanthara, ArunWang, PiwenKang, HeeWang, QunKang, Heon-joongWang, San-LangKaragouni, Amalia D.Wang, Shang-kweiKarapanagiotidis, Ioannis T.Wang, Tzu-WeiKashman, YoelWang, WeiKatsifas, Efstathios A.Wang, Wei-KuangKawai, ShigeyukiWang, Wei-LungKawamura, AkiraWietz, MatthiasKerr, WilliamWioleta, KowalczykKhachik, FrederickWitczak, ZbigniewKhadka, ManojWitulski, BernhardKhokhlatchev, AndreiWlodawer, AlexanderKhozin-Goldberg, InnaWu, Chang-JerKidoaki, SatoruWu, Chieh-HsiKijjoa, AnakeWu, Chin-ChungKikuchi, HaruhisaWu, HongfuKim, DuwoonYamada, ShuheiKim, LeungYamada, SohsukeKiss, RobertYamada, TakeshiKitatani, KazuyukiYamashita, Mari YotsuKlettner, AlexaYamazaki, HiroyukiKobayashi, ToyoharuYan, NingKogure, NoriyukiYang, JyisyKolmar, HaraldYarotskyy, ViktorKomaki, HisayukiYoung, HowardKong, In-sooYoung, WayneKridel, Steven J.Younis, RatherKuan, Yu-HsiangYoza, Brandon A.Kuepper, FrithjofYuan, ZhihongKunanek, JuliaYue, PatrickYingKitKuo, Po-LinZaferanloo, BitaKuo, Tzong-FuZaharof, DavidKurihara, HideyukiZhang, FanKurtán, TiborZhang, FumingKuyucak, SerdarZhang, WenKwak, Jong-YoungZhang, YifanKwon, Il-KeunZhou, Yu-DongKyzas, GeorgeZhu, MikeLaatsch, HartmutZubía, Eva

